# Molecular composition and distribution of gap junctions in the sensory epithelium of the human cochlea—a super-resolution structured illumination microscopy (SR-SIM) study

**DOI:** 10.1080/03009734.2017.1322645

**Published:** 2017-05-17

**Authors:** Wei Liu, Hao Li, Fredrik Edin, Johan Brännström, Rudolf Glueckert, Annelies Schrott-Fischer, Matyas Molnar, Dirk Pacholsky, Kristian Pfaller, Helge Rask-Andersen

**Affiliations:** a Department of Surgical Sciences, Head and Neck Surgery, Section of Otolaryngology, Department of Otolaryngology, Uppsala University Hospital, Uppsala, Sweden;; b Department of Immunology, Genetics and Pathology, The Rudbeck Laboratory, Uppsala University, Uppsala, Sweden;; c Department of Otolaryngology, Medical University of Innsbruck, Innsbruck, Austria;; d Science for Life Laboratory, BioVis Facility, Uppsala University, Uppsala, Sweden;; e Department of Histology and Molecular Cell Biology, Institute of Anatomy and Histology, Medical University of Innsbruck, Innsbruck, Austria

**Keywords:** Cochlea, confocal microscopy, connexin 26/30, human, SR-SIM

## Abstract

**Background:**

Mutations in the GJB2 gene, which encodes the Connexin26 (Cx26) protein, are the most common cause of childhood hearing loss in American and European populations. The cochlea contains a gap junction (GJ) network in the sensory epithelium and two connective tissue networks in the lateral wall and spiral limbus. The syncytia contain the GJ proteins beta 2 (*GJB2*/Cx26) and beta 6 (*GJB6*/Cx30). Our knowledge of their expression in humans is insufficient due to the limited availability of tissue. Here, we sought to establish the molecular arrangement of GJs in the epithelial network of the human cochlea using surgically obtained samples.

**Methods:**

We analyzed Cx26 and Cx30 expression in GJ networks in well-preserved adult human auditory sensory epithelium using confocal, electron, and super-resolution structured illumination microscopy (SR-SIM).

**Results:**

Cx30 plaques (<5 μm) dominated, while Cx26 plaques were subtle and appeared as ‘mini-junctions’ (2–300 nm). 3-D volume rendering of Z-stacks and orthogonal projections from single optical sections suggested that the GJs are homomeric/homotypic and consist of assemblies of identical GJs composed of either Cx26 or Cx30. Occasionally, the two protein types were co-expressed, suggesting functional cooperation.

**Conclusions:**

Establishing the molecular composition and distribution of the GJ networks in the human cochlea may increase our understanding of the pathophysiology of Cx-related hearing loss. This information may also assist in developing future strategies to treat genetic hearing loss.

## Introduction

Gap junctions (GJs) form intercellular channels connecting adjacent cells, allowing the passage of small molecules (<1 kD), ions, metabolites, and electrical impulses. GJ plaques are composed of closely gathered hemi-channels composed of connexin (Cx) proteins arranged as hexameric rings or connexons with a central pore. These plaques are thought to play multiple roles in fluid homeostasis, metabolic supply, nerve excitation, and intercellular Ca^2+^ signaling (1,2).

The human cochlea contains an astonishing number of GJ channels in both epithelial and connective tissue networks. The organization and functions of these networks are poorly understood. The GJ networks are crucial for normal hearing (3), and in the lateral wall they have been associated with the generation of the endocochlear potential (EP) (4–8). Endolymph has a high K^+^ concentration important for hair cell transduction. K^+^ recycling from hair cells is assumed to be mediated by GJs, which allow the passage of K^+^ to the spiral ligament, where it is taken up by fibrocytes (9,10) and relayed via GJ syncytia to the stria vascularis (SV) through electrochemical gradients (11–13). Recently, it was shown that GJs are important for cochlear amplification (14), that miRNAs may pass through GJs, and that Cx-mediated intercellular communication may be required for cochlear development (15,16).

Mutations in the genes encoding Cx26 (*GJB2*) and Cx30 (*GJB6*) cause non-syndromic inherited deafness (3,17–22), and alterations in the GJB2 gene are the most common etiology of childhood hearing loss in American and European populations (17). A review describing the mechanisms underlying Cx mutation-induced hearing loss was recently published by Wingard and Zhao (23).

**Figure 1.**
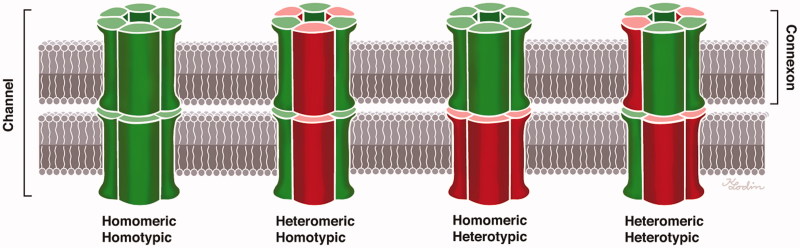
Composition of gap junctions as adapted from Kumar and Gilula (1996) (64). In humans, there are more than 20 isoforms encoded by the gene family, and different cell types may express several connexin isoforms. The possible combinations of hetero-oligomeric connexons seem to be restricted to members of the same subgroup, such as α and β.

Connexons can be homomeric, i.e. consisting of a single connexin isotype, and two identical homomeric connexons can come together to form a homotypic GJ channel. Heteromeric hemi-channels and heterotypic GJ channels have also been described. Hence, different Cx proteins may be present in the same hemi-channel or channel (Figure 1). The function of the GJs may vary with the molecular arrangement and composition of Cx proteins, and multiple different configurations have been described in the cochlea. Co-immunoprecipitation has been used to demonstrate the oligomerization of Cx26 and Cx30 (24–26), indicating that some GJ subunits are heteromeric/heterotypic (27). Cochlear hybrid GJ channel configurations were first described by Zhao and Santos-Sacchi (28) based on patch clamp recordings, and their permeability was first described by Zhao (29). Dye-selective permeation experiments have indicated the presence of Cx26-only channels in supporting cells within the sensory epithelium (27). According to Zhao and Yu (2), Cx26 labeling in the guinea pig cochlear sensory region largely overlaps that of Cx30, but there are also areas of exclusive expression. According to Lautermann et al. (30,31), who used immunofluorescence staining and western blot analyses, Cx30 is the main isoform expressed in the cochlea. In a study of the human organ of Corti using confocal immunohistochemistry, Liu et al. (32) found Cx26/30 co-labeling in the supporting cell area but also found areas of isolated Cx26 or Cx30 expression, suggesting the existence of both homomeric/homotypic and hybrid forms (heteromeric or heterotypic).

A super-resolution structured illumination microscopy (SR-SIM) study on human cochlear material suggested recently that Cx26 and Cx30 proteins are not co-expressed in the lateral wall of the human cochlea but, rather, form closely associated GJ plaques (33). As variations in molecular organization may reflect unique functions, structural characterization is crucial for understanding cochlear physiology and the consequences associated with Cx mutations. Human studies are demanding because well-preserved tissue is difficult to obtain. Moreover, human inner ear tissue is surrounded by the hardest bone in the body. Here, we prepared samples of decalcified human cochleae for SR-SIM, in combination with scanning electron microscopy (SEM) and transmission electron microscopy (TEM), focusing on the sensory epithelium. Tissue was obtained during surgery after patients had provided informed consent. The volume resolution of 3-D SR-SIM is approximately eight-fold higher than that of conventional microscopy (34), with a two-fold improvement in lateral resolution (100–130 nm).

### Materials and methods

The use of human materials was approved by the local ethics committee (no. 99398, 22/9 1999, cont., 2003, Dnr. 2013/190), and patient consent was obtained. The use of animal cochlear material was also approved by the local ethics committee (no. C254/4, C209/10). The study adhered to the guidelines of the Helsinki Declaration.

### TEM

Two archival specimens collected during surgery and two specimens collected after perilymphatic perfusion were analyzed in Uppsala (35,36) and Innsbruck. The specimens were fixed in 3% phosphate-buffered glutaraldehyde, pH 7.4, and rinsed in cacodylate buffer, followed by fixation with 1% osmium tetroxide at 4 °C for 4 h. The specimens were infiltrated with Epon resin in a vacuum chamber for 4 h. For TEM analysis, sections were viewed under a JEOL 100 SX electron microscope (Uppsala) and under Zeiss LIBRA (Institute of Zoology, Innsbruck) and Philips CM 120 (Division of Anatomy, Histology and Embryology, Innsbruck) transmission electron microscopes (Innsbruck).

### Fixation and sectioning of human cochlea for immunohistochemistry

Five cochleae from five adult patients (2 male, 3 female; aged 40–65 years; Table 1) were dissected out as a whole piece during petro-clival meningioma surgery. In the operating room, the cochleae were immediately placed in 4% paraformaldehyde diluted with 0.1 M phosphate-buffered saline (PBS, pH 7.4). After a 24-h fixation period, the fixative was replaced with 0.1 M PBS and then with 10% EDTA solution at pH 7.2 for decalcification. After approximately four weeks, the thoroughly decalcified cochleae were rinsed with PBS. For frozen sections, the cochleae were embedded in Tissue-Tek (OCT Polysciences), rapidly frozen, and sectioned into slices 8–10 μm thick using a Leica cryostat microtome. The frozen sections were collected onto gelatin/chrome alum-coated slides and stored below −70 °C before processing for immunohistochemistry.

**Table 1.** Patient data and method of analysis.

Age (years)	Sex	PTT	Analysis
43	female	50 dB (1 kHz to 8 kHz)	immunohistochemistry
51	male	normal	immunohistochemistry
72	male	50 dB (2 kHz to 4 kHz)	immunohistochemistry
67	female	normal	immunohistochemistry
60	male	normal	TEM
65	male	normal	TEM

dB: decibels; PTT: pure tone threshold; TEM: transmission electron microscopy.

### Antibodies and immunohistochemistry

The Cx30 antibody was a rabbit polyclonal antibody (catalog number 71-2200, Invitrogen, Carlsbad, CA, USA). Its selectivity for human Cx30 was confirmed by western blotting. The anti-Cx26 monoclonal antibody was derived from mice and has a high specificity (1:50, catalog number 33-5800, Invitrogen, Carlsbad, CA, USA). The antibody against laminin β2 was a rat monoclonal antibody (catalog number 05-206, Millipore, Billerica, MA, USA; dilution 1:100) specific for the laminin β2 chain. This antibody was used to demarcate the basal lamina at the bottom border of the epithelium in the organ of Corti (OC). It recognizes and is specific for the laminin β2 chain. The anti-laminin antibody showed no cross-reaction with other basement membrane components, such as type IV collagen. Both a polyclonal antibody (catalog number 04-1049, Millipore, Billerica, MA, USA; dilution 1:200) and a monoclonal tubulin antibody (catalog number MAB1637, Millipore, Billerica, MA, USA; dilution 1:200) against neuron-specific class III beta-tubulin (Tuj-1) were used. The combinations, characteristics, and sources of antibodies used in this study are summarized in Table 2. The immunohistochemistry procedures performed on cochlear sections have been described in previous publications (Liu et al. 2009, 2016). Briefly, sections on slides were incubated with an antibody solution under a humid atmosphere at 4 °C for 20 h. After rinsing with PBS (3 × 5 min), the sections were incubated with secondary antibodies conjugated to Alexa Fluor 488 and 555 (Molecular Probes, Carlsbad, CA, USA), counter-stained with the nuclear stain DAPI (4’,6-diamidino-2-phenylindole dihydrochloride) for 5 min, rinsed with PBS (3 × 5 min), and mounted with Vectashield (Vector Laboratories, Burlingame, CA, USA) medium. Primary and secondary antibody controls and labeling controls were prepared to exclude endogenous labeling or reaction products (37). Control sections were incubated with 2% bovine serum albumin (BSA) without the primary antibodies. As a result, the control slides showed no visible staining in any cochlear structures. Both wide-field and confocal fluorescent imaging software employed sensitive fluorescent saturation indicators to prevent overexposure.

**Table 2.** Antibodies used in this investigation.

Targeting protein	Type	Dilution	Host	Catalognumber	Company
Laminin β2	monoclonal	1:100	rat	#05-206	Millipore
Type II col	monoclonal	1:100	mouse	CP18	Millipore
Cx30	polyclonal	1:100	rabbit	71-2200	Invitrogen
Cx26	monoclonal	1:50	mouse	33-5800	Invitrogen
Cx26	polyclonal	1:200	rabbit	ACC-2121	Alomone
Tuj-1	polyclonal	1:200	rabbit	#04-1049	Millipore
Tuj-1	monoclonal	1:200	mouse	MAB1637	Millipore

### Imaging and photography

The stained sections were visualized with an inverted fluorescence microscope (Nikon TE2000) equipped with a spot digital camera with three filters (emission spectrum maxima at 358, 461, and 555 nm). Image-processing software (NIS Element BR-3.2, Nikon) including image merging and fluorescence intensity analyzer features was installed on a computer system connected to the microscope. For laser confocal microscopy, we used the same microscope equipped with a three-channel laser emission system. The optical scanning and image-processing tasks, including the reconstruction of Z-stack images into projections and 3-D images, were performed using Nikon EZ-C1 (ver. 3.80) software. Structured illumination microscopy (SR-SIM) was performed with a Zeiss Elyra S.1 SIM system using a 63x/1.4 oil Plan-Apochromat lens (Zeiss), a sCMOS camera (PCO Edge), and ZEN 2012 software (Zeiss). Multicolor SR-SIM imaging was achieved with the following laser and filter setup: first channel, 405-nm laser excitation and BP 420-480 + LP 750 filter; second channel, 488-nm laser excitation and BP 495-550 + LP750 filter; and third channel, 561-nm laser excitation and BP 570-620 + LP 750 filter. To maximize image quality, five grid rotations and five phases were used for each image plane and channel. The grid size was automatically adjusted by the ZEN software for each excitation wavelength. SR-SIM images were processed with ZEN software using automatic settings and theoretical point spread function (PSF) calculation. A 3-D reconstruction was performed from the SR-SIM dataset using Imaris 8.2 software (Bitplane, Zürich, Switzerland). The microscope was capable of achieving a lateral (*X–Y*) resolution of approximately 100 nm and an axial (*Z*) resolution in the approximately 300 nm range (38). The resolution of the SIM system was measured with sub-resolution fluorescent beads (40 nm, Zeiss) in the green channel (BP 495-550 + LP750) at the BioVis facility in Uppsala. An average PSF value was obtained from multiple beads using the built-in experimental PSF algorithm of the ZEN software. The typical resolution of the system was 107 nm in the *X–Y* plane and 394 nm in the *Z* plane (Supplementary Figure 1, available online).

## Results

### Confocal microscopy

**Figure 2.**
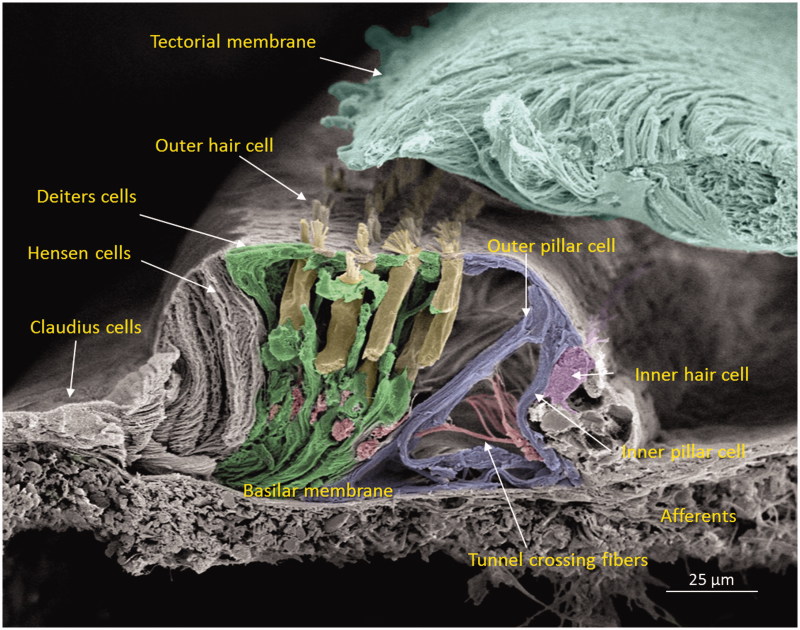
Scanning electron microscopy of the cochlear sensory epithelium (organ of Corti) in the low-frequency region. Modified versions of this image were published earlier in the *Anatomical Record* (65) and with permission in the book *Functional Ultrastructure: Atlas of Tissue Biology and Pathology* by Margit Pavelka and Jürgen Roth (2015) (66).

The human cochlea contains three GJ networks: one in the sensory epithelium and two in the connective tissue (lateral wall/spiral limbus). Figure 2 shows the complex cell architecture in a well-fixed human OC using SEM. Confocal microscopy revealed that Cx30 was highly expressed, while Cx26 was barely detectable. Cx30 staining extended both medially into the inner sulcus and laterally to the outer sulcus and root cells at the spiral prominence.

### SR-SIM

**Figure 3.**
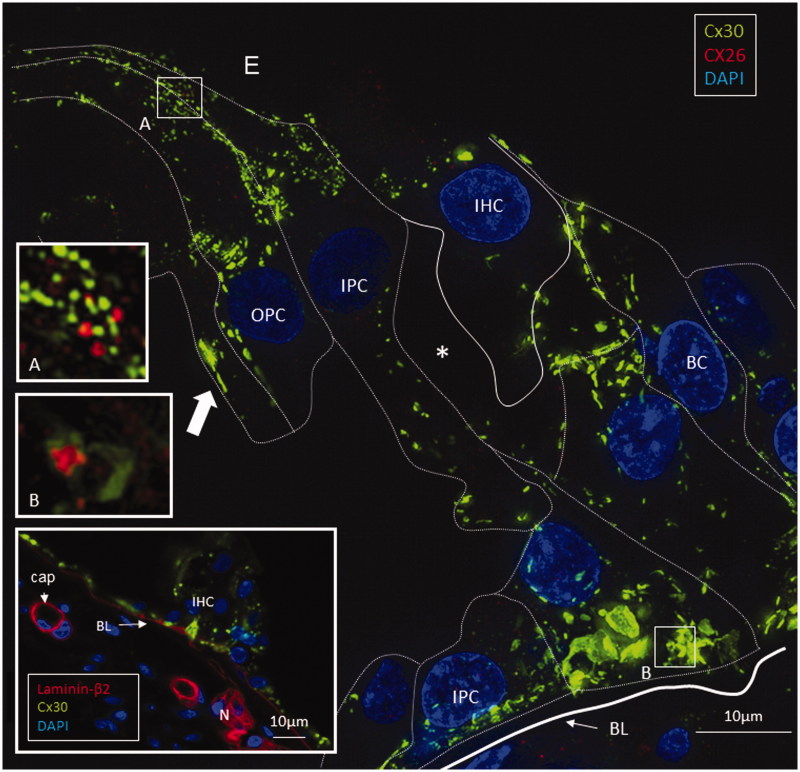
SR-SIM of the inner hair cell (IHC) region in a human cochlea (single optical plane). Cx30 is richly expressed, while Cx26 is hardly detectable. Outer pillar columns display prominent intercellular GJs (filled arrow). Inner pillar feet show large Cx30-positive areas facing the basal lamina (BL). Inset: Confocal micrograph of IHC region. The basal lamina of the epithelium, blood vessels, and neurons are stained for laminin β2. Frames A and B are magnified in insets. A: A few Cx26 GJ plaques are seen among the Cx30-positive GJs. B: An annular GJ with central Cx26-positive domains is seen in the pillar foot. BC: basal cell; E: endolymph; IPC: inner pillar cell; OPC: outer pillar cell.

**Figure 4.**
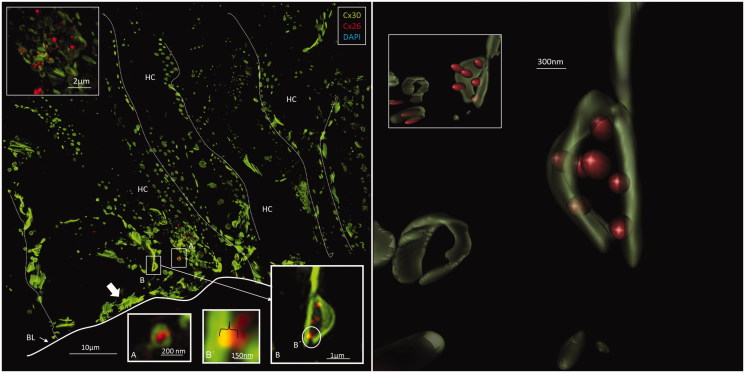
*Left*: A: Maximum-intensity projection of the Hensen cell (HC) region in the human organ of Corti. Cx30-positive intercellular GJ plaques dominate. Some GJs seem to face the BM (filled arrow). Cx26-expressing GJ plaques are located near the Cx30 plaques (inset top left). Framed areas are magnified in insets A and B. Annular GJ plaques with Cx26 expressed inside can be seen. B: An annular GJ surrounds smaller dots of Cx26 staining. One is superimposed (yellow) on the Cx30 plaque and is shown in higher magnification in B. *Right*: A 3-D reconstruction of Cx26 (red) and Cx30 (green) protein expression is shown in B. The green signal was reconstructed in surface rendering mode, and the red signal was rendered in spot detection mode using Imaris 8.2 software. The inset demonstrates the GJ complex after clock-wise rotation. A single optical plane with orthogonal sectioning is shown in the Supplementary material. BM: basilar membrane.

**Figure 5.**
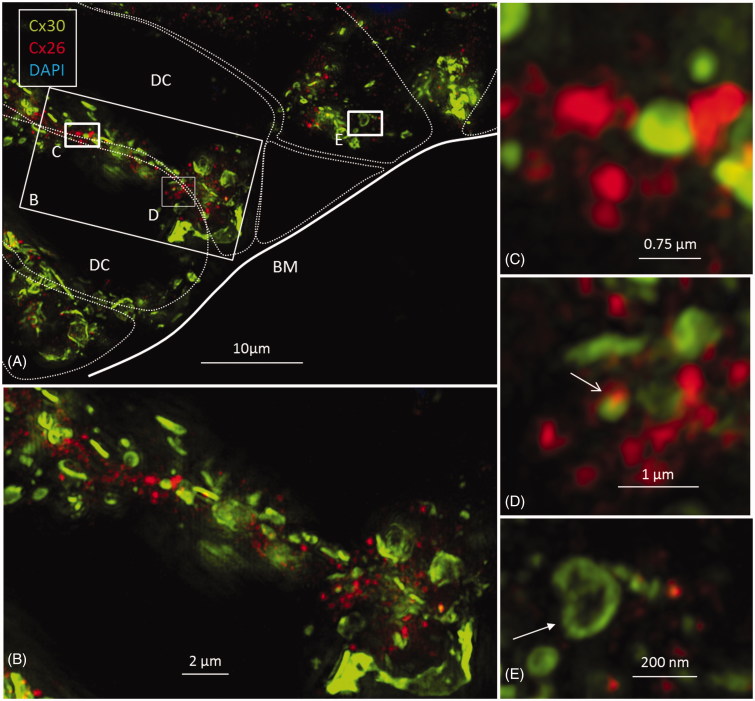
A: Maximum-intensity projection of Cx26 (red) and Cx30 (green) protein expression beneath the outer hair cell region of the human organ of Corti. Cx26 and Cx30 seem to be expressed separately. Cell borders are outlined. Framed areas are magnified in B–E. B and C: Cx26 is mostly expressed as small dots in close association with larger Cx30-positive plaques. D: Superimposed GJs are stained yellow (arrow). E. The Cx30-positive GJ profile (arrow) may reflect degradation and invagination of the channel plaque into the cytoplasm. BM: basilar membrane; DC: Deiters cell.

**Figure 6.**
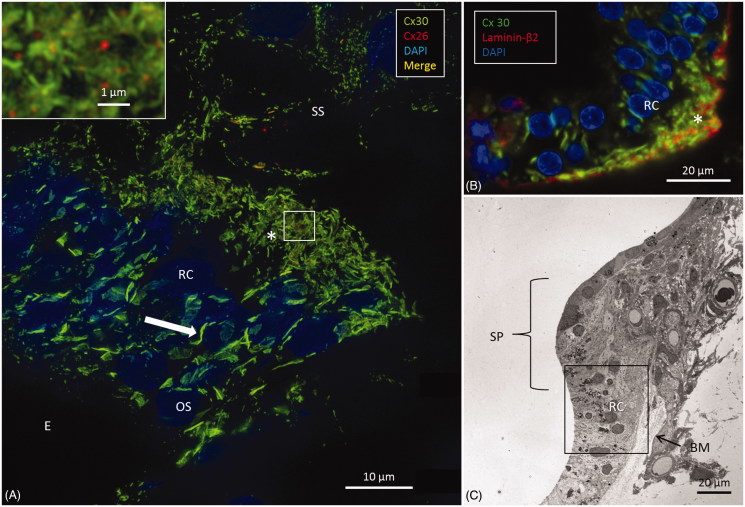
A: SR-SIM of outer sulcus (OS) epithelium and root cells (RCs) at the basal turn of the human cochlea (maximum-intensity projection). Larger Cx30-positive epithelial GJ plaques are seen in the nuclear region (filled arrow). In the basal region of the RCs, large numbers of smaller Cx30-positive plaques are expressed (*). A few Cx26-positive plaques are intertwined among the basal Cx30 plaques (inset of framed area). The sub-epithelial space (SS) also expresses Cx30 in type II fibrocytes. B: Immunofluorescence of laminin/Cx30 co-labeling (*) shows that this network is located between the epithelial root cell processes. C: The corresponding region seen with TEM. BM: basilar membrane; E: endolymph; SP: spiral prominence.

Isolated scans, 3-D rendering, and maximum-intensity projections (MIPs) were analyzed and compared. Cx26 was weakly expressed. Numerous Cx30-positive plaques were found between all supporting cells. The shapes of the Cx30 plaques varied (round, discoid, or elongated), and their size was up to five microns in diameter. In contrast, the Cx26 plaques were small (0.1–0.5 μm) and dot-like (Figures 3–6). No labeling was observed between the hair and supporting cells. The geometry of the Cx30 plaques varied in different supporting cells. Between Hensen cells (HCs) and Deiters cells (DCs), the plaques were round, ovoid, and disc-like and increased in size from the apical surface to the base of the cells (Figure 4, left; Figure 5). Smaller Cx30 plaques were observed between the inner and outer pillar heads along with a few Cx26 ‘mini-plaques’ (Figure 3, inset A). Prominent Cx30-positive plaques or stripes were observed between outer pillar columns (Figure 3). The inner pillar feet displayed prominent, irregularly shaped plaques. A few supporting cell plaques faced the basal lamina. The lateral cell membrane of the outer pillar columns showed extensive Cx30 GJ stripes (Figure 3).

### Association of Cx26 and Cx30 labeling

Cx26 staining was mostly independent of Cx30. Although some areas showed superimposed Cx26 and Cx30 staining, orthogonal views of single optical sections showed separate color signals (Supplementary Figure 2, available online). Annular patterns of Cx30 staining were frequent. These rings had a diameter of 100–1000 nm, with a central subdomain composed of Cx26 (Figure 4, left, insets). A 3-D reconstruction of Cx26 and Cx30 expression is shown in Figure 4. The green signal was reconstructed in surface-rendering mode, and the red signal was rendered in spot detection mode using Imaris 8.2 software. Neurons beneath outer hair cells (OHCs) were positive for the neuron marker Tuj-1 but not for Cx26 or Cx30 (Supplementary Figure 5 inset, available online). Nerve endings beneath the inner hair cells (IHCs) were generally swollen and did not show any staining.

### Outer sulcus epithelium and root cells

Epithelial cells in the outer sulcus heavily expressed Cx30. The root cells displayed two separate domains of GJ plaques. Large Cx30 plaques were seen at the nuclear level, whereas on the basal surface, facing the sub-epithelial space, plaques were smaller (<1 μm) and associated with Cx26 plaques (Figure 6(A), inset). Laminin β2 co-staining of the basal lamina (BL) confirmed that these GJ plaques were located between the root cell processes (Figure 6(B)). Across the BL, the type II fibrocytes also exhibited numerous Cx30-positive GJs. These plaques were similar in size to those between the root processes and were occasionally associated with a few Cx26-positive GJ plaques (Figure 6).

### Transmission electron microscopy (TEM)

TEM images confirmed the copious number of GJ plaques existing between supporting cells. Border and inner phalangeal cells (IPhCs) surrounding the IHCs displayed large numbers of GJs (Supplementary Figure 3, available online). In some cases, folded IPhCs were squeezed between an IHC and an inner pillar cell (IPC). The presence of GJs could not be verified with certainty between the IPC and IPhC (Supplementary Figure 3(A,B), available online). No GJs were observed between sensory and supporting cells. The opposing lateral cell membranes of the Claudius cells (CCs) often had an undulating outline decorated with GJs (Supplementary Figure 4, available online). Annular GJs were not observed with TEM. The lateral cell membranes between HCs and Boettcher cells showed occasional sub-plasma membrane sacs suggestive of rough ER (not shown). The basal plasma membrane of the supporting cells, facing the basilar membrane (BM), often displayed focal densities (Supplementary Figure 5, available online). On confocal microscopy and SR-SIM, these sites frequently demonstrated Cx30 positivity, suggesting that they represented hemi-junctions (Figure 3, inset). The lateral cell membranes of the basal components of the DCs and IPCs were tightly assembled and had several intercellular GJs. The presence of an extensive number of GJs between the basal processes of the root cells was also confirmed with TEM.

## Discussion

### Subtle Cx26 expression in the adult human organ of Corti

There was surprisingly little expression of Cx26 in the sensory epithelium compared to Cx30. However, a fairly large amount of diffuse intracellular Cx26 staining was observed in DCs, particularly near the base of OHCs. This finding suggests that the epithelial GJ network consists mostly of Cx30 in human adults. MIP results indicated possible co-expression, but 3-D rendering revealed that Cx26 and Cx30 were expressed separately in different plaques. These findings indicate that GJs consist mainly of homomeric/homotypic Cx pairs (Figure 4). A similar arrangement was found in the lateral wall of the human cochlea (33). This result was unexpected, as the selective vulnerability of the ear to GJB2 gene disruption has been linked to unique oligomeric varieties of heteromeric GJs, as found in several other tissues (21,26). The molecular and subunit composition of GJs influences their physiological properties and permeability characteristics (39), including both the speed and type of molecular passage (e.g. intercellular Ca^2+^ signaling), which can differ between heteromeric and homomeric GJs (26). Heteromeric channels show selective biochemical properties (28,29). According to Sun et al. (26), hybrid Cx26/30 cochlear GJs show faster intercellular Ca^2+^ signaling than the homomeric forms. These authors also found that Cx26 and Cx30 co-localize in most GJ plaques in the cochlea in co-immunoprecipitation experiments. In the present analysis, the Z-stacks that were converted into MIPs to create a 2-D image lacked depth information in the *Z* plane. Therefore, 3-D renderings were prepared from the Z-stacks to discriminate between objects on the *Z*-axis, and single optical sections in orthogonal planes enabled the accurate visualization of the physical relationship between Cx26 and Cx30 GJ plaques (Figure 4; Supplementary Figure 2, available online). The close relationship between Cx26 and Cx30 homomeric/homotypic GJ plaques suggests a yet-undefined functional link and cooperation.

### Cx30 and motility of supporting cells

The widespread distribution of Cx30-positive GJ plaques suggests that they are crucial for human hearing. The GJ syncytium connecting most supporting cells may supply the avascular sensory epithelium with nutrients as well as remove metabolic waste products from the highly active hair cells. The outer pillar columns also showed lateral intercellular Cx30 strands. Such coupling may be important to synchronize the motion of these cells, allowing them to act in concert for cochlear amplification (14). GJ strands were not seen between the inner pillar columns, suggesting that these cells have different biophysical properties. The inner pillars are anchored to a static foundation, whereas the outer pillars are tightly connected to the mobile BM.

### K + recycling—spectacular Cx30 network at the root cells

Various models have been presented for the transfer of K^+^ ions from the sensory epithelium back to the connective tissue GJ networks in the lateral wall (10,40–44). These transfer mechanisms include both a medial and a lateral trans-epithelial flux of K^+^ as well as extra-epithelial recycling to the perilymph (40,41). An extra-epithelial K^+^ recycling system would involve a basally directed flow across the cell membrane and the extra-cellular matrix of the BM into the scala tympani. Our findings support the existence of medial and lateral trans-cellular K^+^ recycling across Cx30 GJs. The multitude of GJs between root cell processes and the type II fibrocytes was remarkable (Figure 6). The outer sulcus cells and root cells exhibited two different GJ systems closely related to the sub-epithelial GJ network. Intense labeling for Cx26 and Cx30 in the outer sulcus cells and root processes was previously demonstrated by Liu and Zhao (44). Additionally, it has previously been proposed that K^+^ ions are transferred through rectifying K^+^ currents at the basolateral processes of root cells, likely mediated via Kir4.1 channels (10). The Kir4.1 channels have also been found to co-localize with aquaporin channels (42) to maintain hydrostatic equilibrium within the spiral ligament micro-environment. These results seem highly suggestive of an active radial ion flux across these cell layers.

### Mechanisms underlying Cx deficiency indeafness—recent findings

Until recently, deafness caused by GJB6 (Cx30) deletion was believed to be due to defective Cx26 expression, and Cx30 was considered dispensable for cochlear function (45). According to Schütz et al. (46), it may be difficult to isolate the role of Cx30 from that of Cx26 because the experimental alteration of Cx30 causes a downregulation of Cx26. The two genes share almost 80% identity at the protein level. Human deletion mutations in both Cx26 and Cx30 cause deafness (3,20,47).

Considering the important role played by Cx26 in human deafness, there is a discrepancy between embryonic and adult human tissue that may be explained by a maturation process. The development of the cochlear GJ system precedes the functional maturation of the rat inner ear that takes place between the second and third postnatal weeks (30). Kamiya et al. (48) suggested that Cx26 may be crucial for large GJ plaques to form during embryonic development and for the establishment of sensory function but less important for the maintenance of the mature OC. Previous results have also indicated that GJB2 mutations disturb the homeostasis of the extra-cellular space surrounding the sensory hair cells rather than endolymph homeostasis. Impaired K^+^ transport by supporting cells may lead to a degeneration of the OC (49). These changes were shown to occur at a very early stage of development (50). Therefore, Cx26 may be more vital for the maturation of the sensory epithelium (51,52) and less necessary for normal hearing, while Cx30 may be essential for normal repair following sensory cell loss (53,54). Mouse models demonstrate that Cx26 mutations can cause both congenital deafness and late-onset, progressive hearing loss through different mechanisms. Congenital deafness was thought to be the result of cochlear mal-development, whereas late-onset hearing loss was associated with reductions in cochlear active amplification, which is dependent on supporting cell GJs (14,55). GJB2 deletion of Cx26 before, but not after, postnatal day 5 caused congenital deafness due to a closed cochlear tunnel (56). Deafness was not due to EP reduction (57). The authors concluded that K^+^ recycling may not be a deafness mechanism for GJ deficiency-induced hearing loss in these animals (23).

### ‘Hybrid plaques’

**Figure 7.**
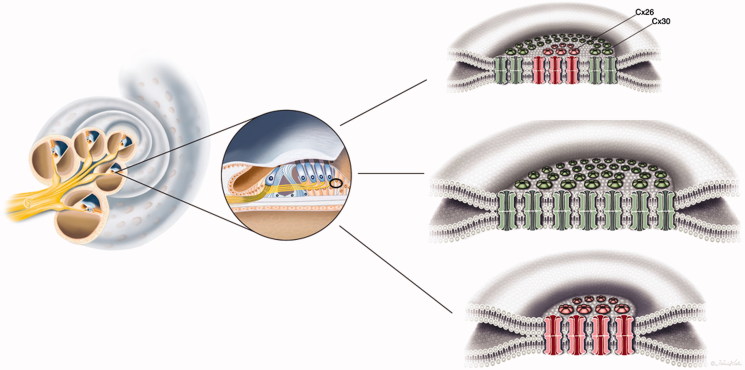
Illustration of gap junction (GJ) plaques in the human organ of Corti. GJ plaques may exist as Cx26 or Cx30 protein homomeric/homotypic aggregates. Some GJ plaques appeared to contain both isotypes. From this experiment, it was not possible to verify whether single plaques consisted of both molecular arrangements (‘hybrid plaque’) or whether separate homomeric GJ plaques were just in close juxtaposition.

Remarkably, Cx26 subdomains appeared inside annular Cx30 plaques (Figure 3, inset B; Figure 4). Cx proteins have a half-life of only hours (58), reflecting their participation in extremely dynamic physiological processes. This activity is corroborated by the involvement of sub-membrane-localized rough ER in the production and swift membrane incorporation of these proteins, as described in Cx43-GFP-transfected HeLa cells (59). Similar organelles were found between Hensen and Boettcher cells (not shown). According to Jordan et al. (60), who generated time-lapse video of live cells, the entire GJ or a fragment thereof can be internalized into one of the two opposing cells as an annular junction during GJ turnover. Some of the annular GJ plaques identified here may not represent degradation, as they had a central subdomain and were not recognized on TEM. 3-D reconstruction showed that the two categories of GJs were separate but physically interrelated. This distinction indicates that Cx26 and Cx30 plaques may act in concert, despite performing separate functions. Consequently, we hypothesize that diverse aggregates of GJ channels can populate the same GJ plaque or represent physically interacting plaques (Figure 7). Different Cx isoforms have been reported in well-defined GJ plaques using freeze-fracture replica immunogold labeling (FRIL), allowing the co-localization of different Cx proteins (61).

### Hemi-channels

In the present study, TEM showed membrane densities, and SR-SIM demonstrated Cx30 plaques at the basal surface of the supporting cells facing the BM, which may represent hemi-channels. Hemi-channels facing the extra-cellular tissue have been described in the cochlea (29). Hemi-channels may pass ATP to the extra-cellular compartment so that it can bind to purinergic receptors on neighboring cells and act as a signaling molecule.

In summary, the present results seem to support the notion that GJs in the human cochlear sensory epithelium are homomeric/homotypic and that plaques are mostly populated by assemblies of identical GJs that express either Cx26 or Cx30. The possibility of mixed plaques cannot be excluded as the diameter of each GJ is below the maximal resolution of SR-SIM. Establishing the molecular composition of the GJ networks in the human cochlea is essential for understanding the pathophysiology of Cx-related hearing loss and may also assist in developing future strategies to treat genetic hearing loss (62,63).

## Supplementary Material

Supplemental data
