# Supplementary figures and images for: Molecular composition and distribution of gap junctions in the sensory epithelium of the human cochlea—a super-resolution structured illumination microscopy (SR-SIM) study
Source: Ups J Med Sci. 2017 May 17;122(3):160–70. doi: 10.1080/03009734.2017.1322645 (PMC5649321; doi:10.1080/03009734.2017.1322645)

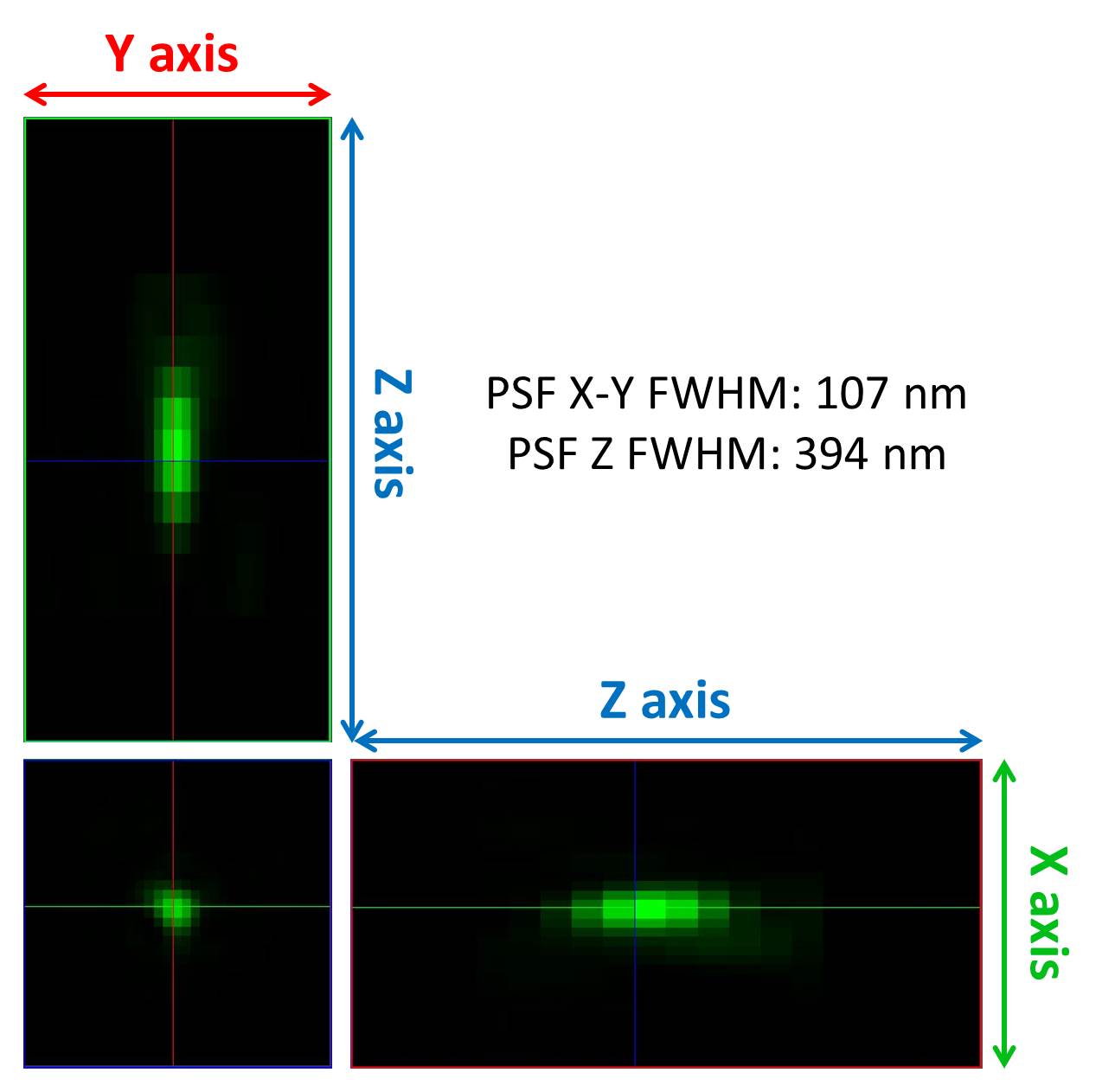

Supplement: Supplemental data [file IUPS_A_1322645_SM8036.zip › 1322645_Supp_Mat/Supplementary material. Figure 1.jpg]

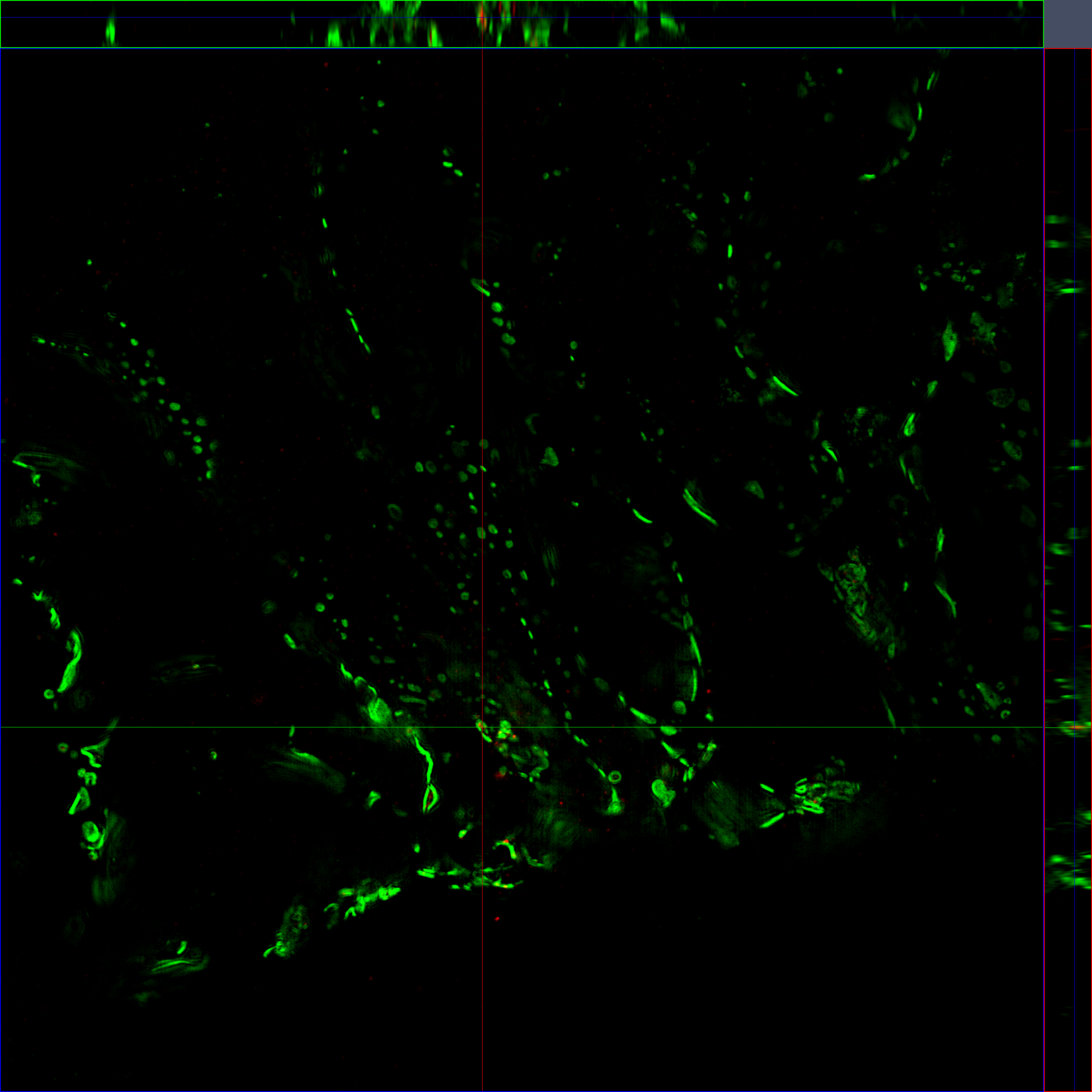

Supplement: Supplemental data [file IUPS_A_1322645_SM8036.zip › 1322645_Supp_Mat/Supplementary material. Figure 2.jpg]

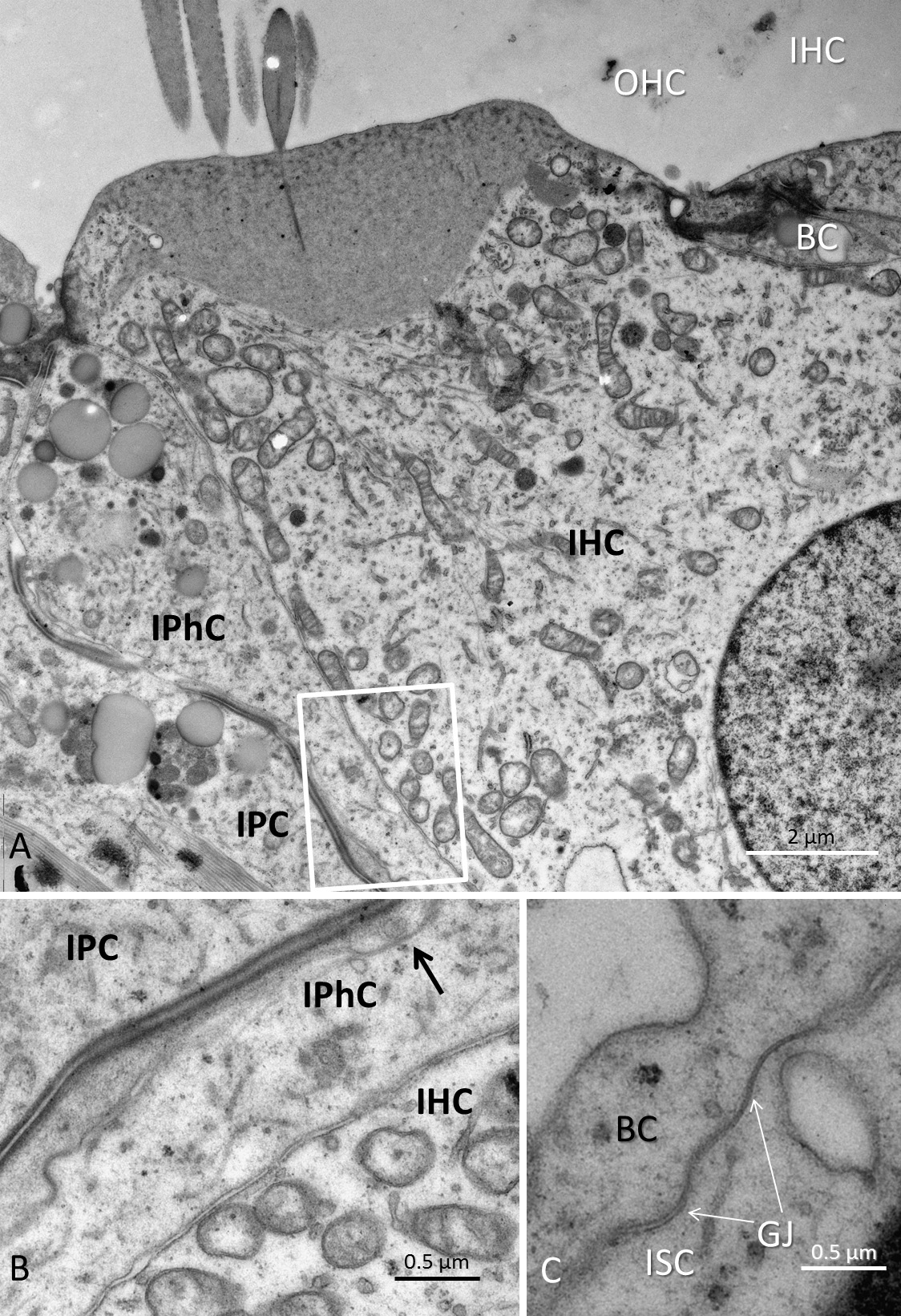

Supplement: Supplemental data [file IUPS_A_1322645_SM8036.zip › 1322645_Supp_Mat/Supplementary material. Figure 3 compressed.jpg]

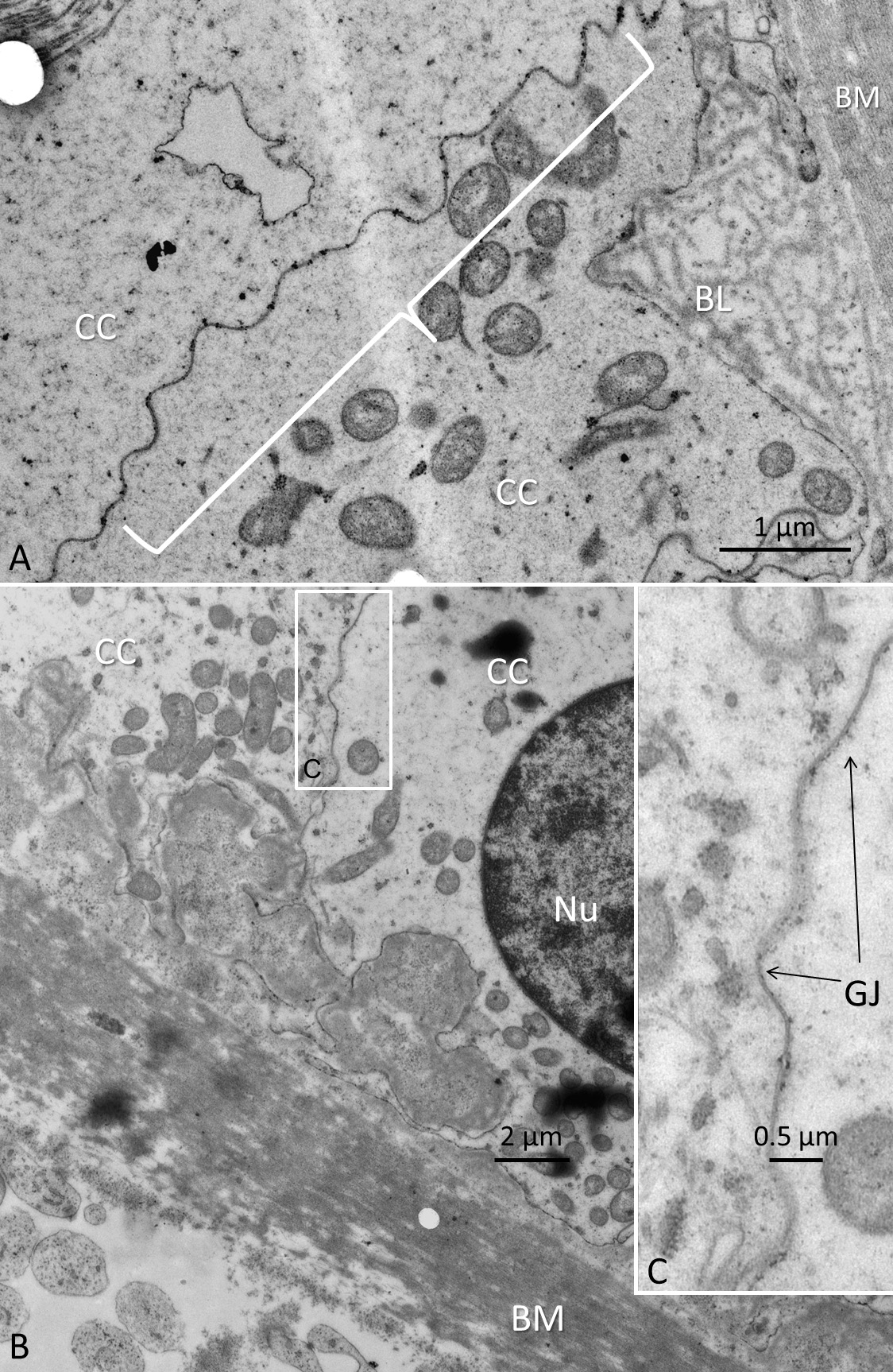

Supplement: Supplemental data [file IUPS_A_1322645_SM8036.zip › 1322645_Supp_Mat/Supplementary material. Figure 4 compressed.jpg]

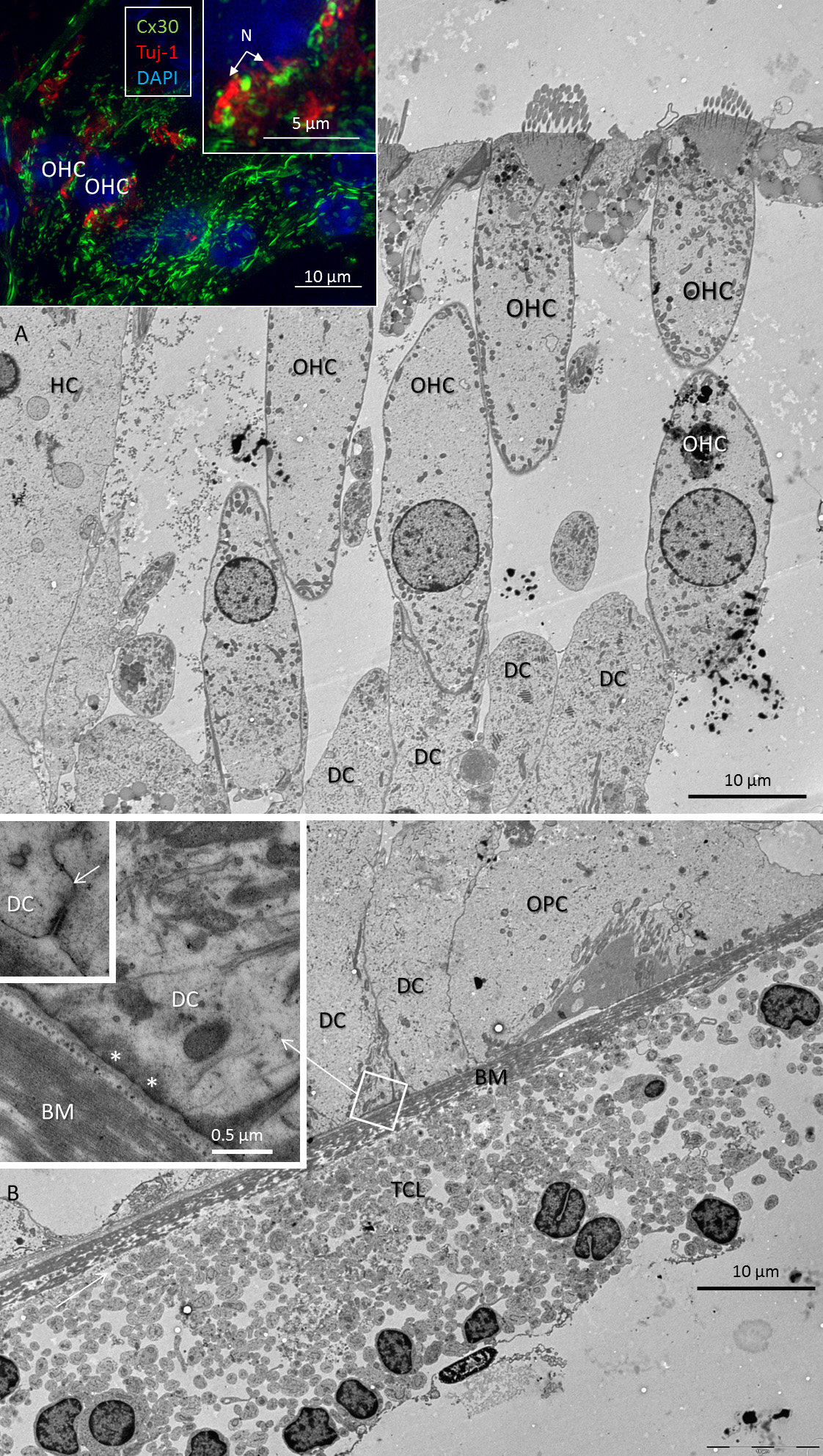

Supplement: Supplemental data [file IUPS_A_1322645_SM8036.zip › 1322645_Supp_Mat/Supplementary material.Figure 5 compressed.jpg]
